# Weak antilocalization in Cd_3_As_2_ thin films

**DOI:** 10.1038/srep22377

**Published:** 2016-03-03

**Authors:** Bo Zhao, Peihong Cheng, Haiyang Pan, Shuai Zhang, Baigeng Wang, Guanghou Wang, Faxian Xiu, Fengqi Song

**Affiliations:** 1 National Laboratory of Solid State Microstructures, Collaborative Innovation Center of Advanced Microstructures, and College of Physics, Nanjing University, Nanjing, 210093, P.R. China; 2 State Key Laboratory of Surface Physics and Department of Physics, Collaborative Innovation Center of Advanced Microstructures, Fudan University, Shanghai 200433, P.R. China

## Abstract

Recently, it has been theoretically predicted that Cd_3_As_2_ is a three dimensional Dirac material, a new topological phase discovered after topological insulators, which exhibits a linear energy dispersion in the bulk with massless Dirac fermions. Here, we report on the low-temperature magnetoresistance measurements on a ~50 nm-thick Cd_3_As_2_ film. The weak antilocalization under perpendicular magnetic field is discussed based on the two-dimensional Hikami-Larkin-Nagaoka (HLN) theory. The electron-electron interaction is addressed as the source of the dephasing based on the temperature-dependent scaling behavior. The weak antilocalization can be also observed while the magnetic field is parallel to the electric field due to the strong interaction between the different conductance channels in this quasi-two-dimensional film.

Dirac materials, with linear band dispersion in low-energy excitation such as graphene and topological insulators, have been receiving increasing attention owing to the possibility of being a new candidate for next-generation electronic and spintronic devices^1^,^2^. Recently, theory predicts that Cd_3_As_2_^ ^3 and Na_3_Bi^4^ are the three dimensional (3D) Dirac materials, soon after which, they have been experimentally demonstrated by angle-resolved photoemission spectroscopy (ARPES)^5^,^6^,^7^, scanning tunneling microscope (STM)^8^ and electrical transport measurements^9^. Remarkably, various novel topological phases, such as Weyl semimetals, topological insulators and topological superconductors can be obtained from 3D Dirac materials by breaking the time reversal symmetry or inversion symmetry^10^,^11^. More importantly, electrical transport measurements of Cd_3_As_2_ bulk crystals exhibit many novel phenomenona, such as high mobility, giant and linear magnetoresistance (MR), non-trivial quantum oscillations and Landau level splitting under magnetic fields^8^,^9^,^12^,^13^. Besides, the superconductivity phenomenon has been evidenced on the surface of Cd_3_As_2_ crystals. It could be topological superconductors^14^. Another important phenomenon is the negative MR which confirms the existence of chirality in Weyl fermions that has been observed in Cd_3_As_2_ nanowires^15^ and nanoplates^16^. And in the systems of Bi_0.97_Sb_0.03_^17^, TaAs^18^,^19^, ZrTe_5_^20^ and Na_3_Bi^21^, the chiral anomaly induced negative MR has also been observed.

In low-dimensional structures, studies of electrical transport have revealed several quantum interference (QI) phenomena at low temperature, including weak localization (WL) or weak antilocalization (WAL) and universal conductance fluctuations (UCF). The physical properties of some materials can be determined from these QI phenomena including the temperature dependence of the resistance, Hall effect, and MR. In 2D films, these contributions are logarithmic in temperature and have nontrivial dependence on the magnetic field. In perpendicular and parallel fields, MR always shows different behavior since orbital QI effects are sensitive to field orientation^22^,^23^,^24^. These techniques are well established but continue to be useful in the study of electrical transport in a variety of systems^23^,^24^,^25^. WAL phenomenon is always observed in Dirac materials, such as topological insulators and graphene without inter-valley scattering, as an important consequence of spin-momentum locking and the full suppression of backscattering, resulting in a relative π Berry phase acquired by electrons executing time-reversed paths^26^,^27^. Recently, the WAL effect has also been observed in 3D Dirac semimetal Cd_3_As_2_^28^,^29^, 3D Weyl semimetal TaAs^18^,^19^ and Bi_0.97_Sb_0.03_^17^. In addition, the WAL effect by theoretical description using the Feynman diagram shows its origin from the inter-valley scattering as described in two-dimensional (2D) Dirac materials such as graphene and topological insulators^30^.

However, the WAL effect is always absent in 2D films when applied parallel magnetic field due to the suppressed interference in a closed electron path. As an effective tool to investigate the scattering ratio in parallel field, the WAL effect is indispensable. In this work, we study the magnetoresistance of ~50 nm-thick Cd_3_As_2_ films. We have found a sharp cusp around 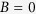 which is stable when the magnetic field is perpendicular to the film (B⊥E) or parallel to electric field (B‖E). We take into account 2D HLN formula in the presence of spin-orbit coupling to discuss the WAL effect in B⊥E while we explore the origin of the WAL effect in B‖E, where a negative MR is also observed.

## Results and Discussion

**Figure 1:**
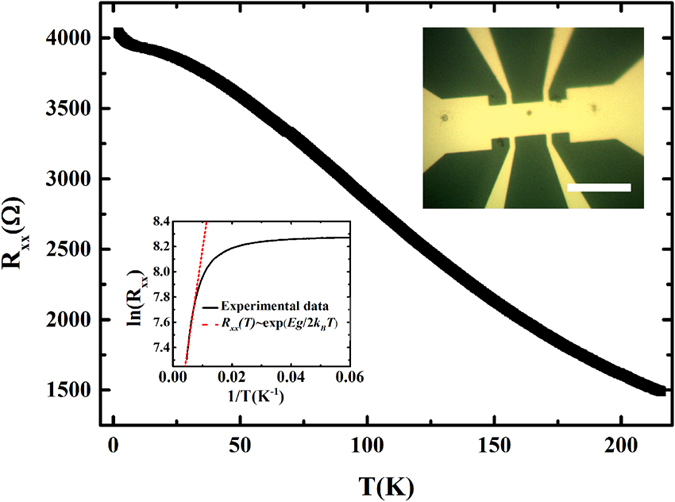
Temperature dependence of the resistance of the Cd_3_As_2_ film device CA2. The right inset shows its optical image with the scale bar of 50 μm. The left inset shows the Arrhenius fitting of 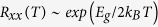
with the result of a 21.9 meV band gap.

Figure. 1 shows the temperature dependence of the resistance 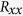 of sample CA2. With the temperature decreasing, 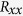 shows an increasing behavior over the temperature range which is different from metallic bulk materials. This insulating behavior is also reported in low-dimensional Cd_3_As_2_ materials^28^,^29^,^31^ and bulk materials under high pressure^32^,^33^. The data can be fitted to the Arrhenius formula of 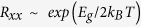 at temperatures from 100 K to 200 K, where 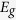 is the bandgap, 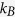 is the Boltzmann constant and 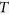 is the measurement temperature. We obtain the band gap 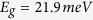, which is reasonable with the value for the Cd_3_As_2_ thin film of this thickness^3^,^29^. When the temperature reaches <100 K, the resistance deviates from the Arrhenius formula and shows weak temperature dependence below 20 K. The sharp increase below 4 K indicates a contribution from electron-electron scattering in the presence of disorder in the 2D films at low temperatures^34^,^35^.

**Figure 2:**
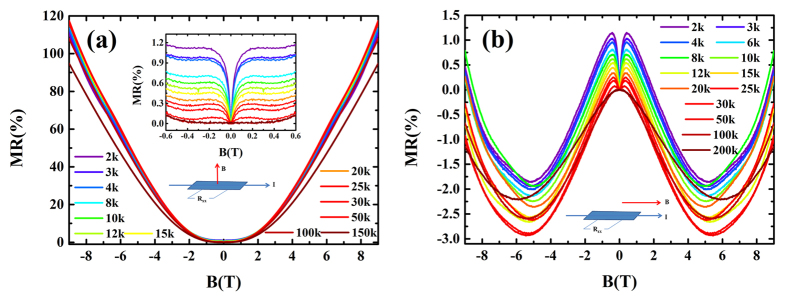
The MR with the applied magnetic field B⊥E or B||E. Schematic diagram of electrical transport measurements are shown in insert respectively. **(a)** The magnetoresistance vs magnetic field in B⊥E at various temperatures from 2 K to 150 K. The insert show the sharp cusp at low magnetic field. **(b)** The magnetoresistance with B‖E at various temperatures from 2 K to 200 K. The WAL effect is also clear around zero fields. With the magnetic field increasing, the MR decreasing shows a negative MR phenomenon with a weak temperature dependent below 150 K. However, it increase with increasing magnetic field when B > 5T.

Figure. 2(a,b) show the MR under applied magnetic fields B⊥E and B‖E respectively at various temperatures. The MR defined as:


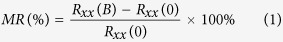


For the perpendicular field of 9T, the MR is around 100% with a weak temperature dependent below 150 K. This is different from the giant MR in bulk samples^36^. Around 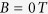, sharp cusps are observed clearly and gradually weaken with increasing temperature. This cusp is ascribed to the quantum interference phenomenon, weak antilocalization, which is also observed in B‖E shown in Fig. 2 (b). However, the MR shows a different behavior in B‖E contrast with that in B⊥E. It decreases with increasing applied magnetic field, showing a negative MR.

Before discussing the magneto-transport properties of the Cd_3_As_2_ films, we first investigate their dimensionality. The film can be treated as a 2D system for the thickness *t* smaller than the appropriate physical length scales. As shown in Table 1, the electronic mean-free path 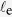 for three samples are obtained with the 2D formula 
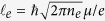
, where 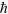 is the Planck constant, 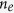 is the 2D carrier density obtained from the Hall effect measurement, *μ* is the carrier mobility extracted from the electronic conductivity formula 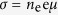. It is clear that the mean-free path 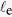 is longer than the thickness *t *= 50 nm for sample CA1 and CA3 and less than the thickness for sample CA2. So the classical diffusive transport is quasi-2D and the treatment before is reasonable. For QI effects, the relevant length scale is the dephasing length *L*_φ_. In nonmagnetic weak disorder systems, the dephasing length is always dominated by inelastic scattering, such as electron-phonon and electron-electron scattering. The electron-phonon scattering with a strong temperature-dependent is always suppressed with decreasing temperature. Thus the *L*_φ_ increases with the temperature decreasing. In the measurement temperature from 100 K to 2 K, 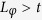 (based on the analysis below) indicates a 2D behavior in the QI range. So we restrict our analysis to this quasi-2D limit.

**Figure 3:**
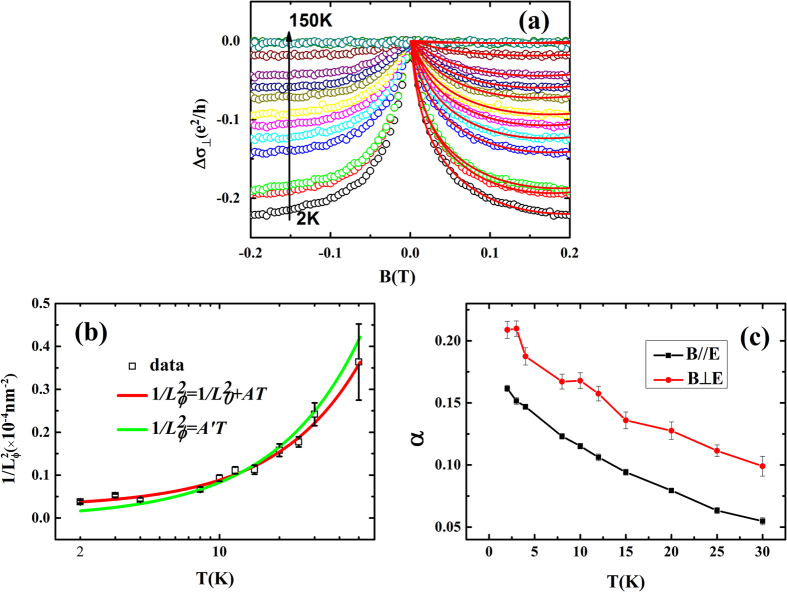
Weak antilocalization effect in applied magnetic field B⊥E. **(a)** The magnetoconductivity of sample CA1 (hole circle) vs magnetic field in B⊥E at temperature from 2 K to 150 K along with fitting (red solid curves) to the Equation 2. (**b**) The dephasing length vs temperature. **(c)** Temperature dependent parameter α with B⊥E or B‖E.

**Figure 4:**
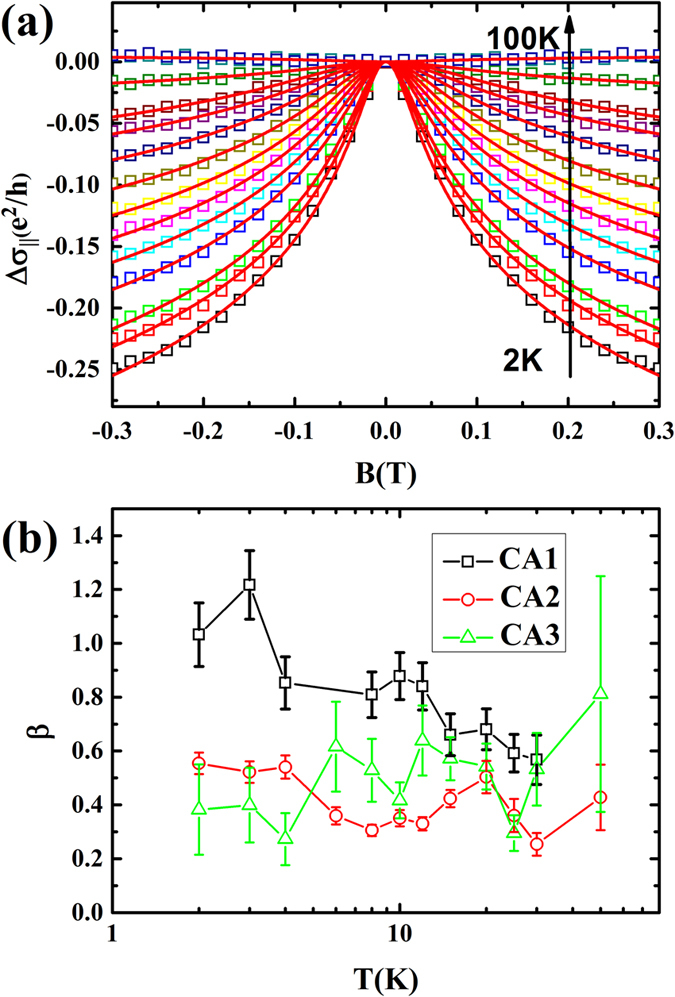
Weak antilocalization effect in applied magnetic field B||E. **(a)** The magnetoconductivity of sample CA1 (hole square) vs magnetic field in B‖E at temperature from 2 K to 100 K along with fitting (red solid curves) to the Equation 3. (**b**) Temperature dependence of β obtained from Equation 3. For sample CA1, the mean free path 64 nm is larger than the film thickness 50 nm.

In Figs 3 and 4, the magnetoconductivity are treated with 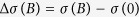 at various temperatures, where 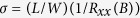, 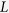 and 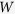 are the length and width of the sample respectively, 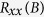 is the resistance under applied magnetic field B⊥E or B‖E.

In 2D systems with an applied perpendicular magnetic field, Hikami, Larkin and Nagaoka (HLN) first described in the presence of spin-orbit coupling for the temperature dependent WAL correction to the conductance^37^. Besides, the background with parabolic conductance contribution is also considered^38^. The correction formula can be written as:


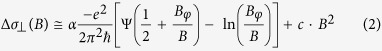


where 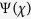 is the digamma function, the characteristic field 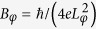, and 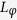 is effective dephasing length. The parameter α takes values of 1/2 and −1 respectively for weak antilocalization and weak localization. The fitting curves for samples CA1 are shown in Fig. 3(a) with red solid curves. The parameter α, shown in Fig. 3(c), with a value of 0.2 at 2 K that is less than it proposed by theory. The temperature dependent of dephasing length 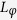 is shown in Fig. 3(b). It is clear that the 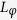 decreases from 513 nm to 110 nm with the temperature increasing from 2 K to 100 K. The extracted 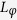 values are larger than the thickness of Cd_3_As_2_ films, justifying the 2D WAL characteristics in our samples.

The parameter α reflects the number of independent conduction channels in the film^26^,^39^. For the 2D electron gas (2DEG) with parabolic dispersion, the magnetoconductivity can transform from WL to WAL (α from −1 to 0.5) as a function of the strength of scattering by spin-orbit impurities. However, for the massless Dirac fermions in 3D topological insulators, the WAL (α = 0.5) always exist for every value of the spin-orbit disorder^40^. Besides, due to the coexistence of topologically trivial 2DEG and topological surface state, the α < 0.5 is observed in 3D topological insulator^41^. For the negative magnetoconductivity in 3D Dirac semimetal, the WAL in low field and short-range scattering in high field are also discussed^30^,^42^. Recently, the Aharonov-Bohm oscillations have been observed in individual single-crystal Cd_3_As_2_ nanowires. It provides transport evidence of the surface state in three-dimensional Dirac semimetals^43^. So the several independent conduction channels may coexist with each other in this Cd_3_As_2_ films with different strength of spin-orbit scattering. It induces α < 0.5.

In the low temperature regime, the dephasing mechanism for 2D system can induce a power-law rule of 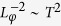 due to the electron-phonon scattering and 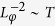 because of the quasielastic Nyquist electron-electron scattering process^44^. Here we proposed the electron-electron interaction and the saturated dephasing mechanism in the Cd_3_As_2_ film and it can be fit with the formula 
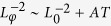
44,45, where 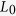 represents the zero-temperature dephasing length. It is sensitive to the impurities and surface scattering. The last term 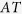 is the contribution from electron-electron interaction. As shown in Fig. 3(b), it results in a perfect fitting with the parameter 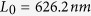. Above all, the electron-electron interaction and the surface scattering or impurities may exist in this film^44^.

Figure. 4(a) shows the WAL effect of the sample CA1 under B‖E. It clearly displays evident cusp around 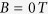 with the 2D character confirmed by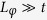. In thin films, Al’tshuler and Aronov (AA) described firstly the quantum corrections to the conductivity when applied magnetic field in the plane of the film^46^ and a similar suppression of QI phenomenon as in the perpendicular field case. The formula for this correction under B‖E can be written as:^24^,^46^


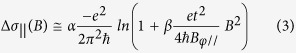


where 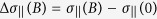. *t* = 50 *nm* is the film thickness. The parameter 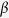 is related to the ratio of mean free path and film thickness. The meaning of the parameter α is described before. However, the value of α = 0.16 in B‖E at 2 K is similar to α = 0.2 in E⊥B, and with a similar temperature dependence in Fig. 3(c). So we assume that the dephasing length in B‖E is equal to the one under B⊥E (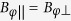)^23^. Thus, the 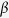 vs temperature can be obtained. The 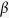 of three samples are shown in Fig. 4(b). For CA1, the value of 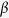 is close to 1 at 2 K and decreases to 0.56 when the temperature increases to 30 K. In this sample, the mean free path 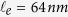 is larger than the film thickness. So this result shows a good agreement with the value of 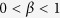 for 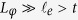 in bilayer system, such as double quantum wells and topological insulators films with two separate surface states^23^,^47^. For CA2 and CA3, it shows a smaller value 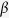 than that in CA1, but it also shows a larger value than the theory proposed with 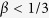 for 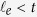 in single layer systems, as shown in Fig. 4 (b).

Although the origin of this obvious WAL effect in this 2D film in B‖E is still a puzzle, a physical origin is proposed to explain this: There are at least two 2D conductance channels contribution in Cd_3_As_2_ films. This field introduces an additional phase for the electron which moves in one channel, then tunnels into another channel, moves there, and finally tunnels back and returns to the initial point.

For B‖E, the MC is upturned with increasing field away from WAL effect range. The experimental observation of this crossover is always attributed to the translation from WAL to WL. The weak localization effect due to the QI is always suppressed by thermal average at higher temperatures. However, for the negative MR in B‖E in Fig. 2(b), it is rather robust and survives at higher temperature. The WL also observed in graphene at room temperature^48^. Besides, this upturn is enhanced in applied magnetic field B‖E. In topological materials, this crossover phenomenon has also been observed at the earliest in 45 nm thick Bi_2_Se_3_ films^49^. It has been attributed to the angle between the spin polarization of surface current and magnetic field direction. Whereas, the Berry phase in this Cd_3_As_2_ films is trivial^29^. Therefore, additional insights will also be required in the future.

## Conclusion

In summary, we have measured the resistance and magnetoresistance of Cd_3_As_2_ films with thickness of ~50 nm under applied magnetic field B⊥E or B‖E. An insulating temperature dependent resistance with a band gap 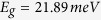 is observed in Cd_3_As_2_ films. Positive MR sharp cusp around 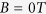 under both applied field were measured, which can be satisfactorily described by existing 2D WAL theory. The electron-electron scattering is suggested as a source of the dephasing mechanism in this Cd_3_As_2_ film. Under B‖E, the WAL effect is also clearly observed and proposed as the strong coupling between the different conductance channels in this quasi-two-dimensional film. The negative MR is observed in B||E in Cd_3_As_2_ films. But the exact origin of this negative MR needs further work.

## Methods

The ~50 nm-thick Cd_3_As_2_ thin films are grown in a molecular beam epitaxy system. The devices are fabricated with standard Hall bar geometry with a metal mask for sample CA1 and ultra-violet (UV) lithography photoresist as mask for CA2 and CA3. The exposed area is etched with argon plasma. The UV photoresist is cleaned with acetone and deionized water. All measurements were carried out at low temperatures down to 2 K with a magnetic field up to 9T. Standard lock-in amplifiers (Stanford Research 830 and 850) were used to acquire data with the electric current of 1 μA. The parameters of all samples are listed in Table 1.

## Additional Information

**How to cite this article**: Zhao, B. *et al.* Weak antilocalization in Cd_3_As_2_ thin films. *Sci. Rep.* **6**, 22377; doi: 10.1038/srep22377 (2016).

## Figures and Tables

**Table 1:** Measured and calculated 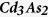 sample parameters.

Device	*L*(μm)	*W*(μm)	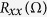	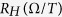	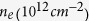	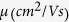	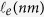
**CA1**	**30**	**80**	**416.8**	**290.36**	**2.15**	**2612.4**	**64**
**CA2**	**25**	**25**	**4039.8**	**991.42**	**0.63**	**2454.1**	**32**
**CA3**	**4**	**4**	**1520.2**	**377.1**	**1.66**	**2480.5**	**53**

The device length is the distance between the two measure voltage probes in a four-probe configuration. The values of 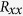, 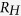, 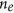, 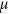, 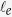 are for 2 K. The longitudinal resistance 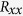, Hall coefficient 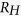 obtained from fitting the Hall resistance with linear curve. The carrier density 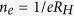, the mobility 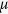 obtained from 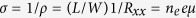, the mean-free path 
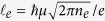
.
